# Spot-Scanning Hadron Arc (SHArc) Therapy: A Study With Light and Heavy Ions

**DOI:** 10.1016/j.adro.2021.100661

**Published:** 2021-02-04

**Authors:** Stewart Mein, Thomas Tessonnier, Benedikt Kopp, Semi Harrabi, Amir Abdollahi, Jürgen Debus, Thomas Haberer, Andrea Mairani

**Affiliations:** a Clinical Cooperation Unit Translational Radiation Oncology, National Center for Tumor Diseases (NCT), Heidelberg University Hospital (UKHD) and German Cancer Research Center (DKFZ), Heidelberg, Germany; b Division of Molecular and Translational Radiation Oncology, Department of Radiation Oncology, Heidelberg Faculty of Medicine (MFHD) and Heidelberg University Hospital (UKHD), Heidelberg Ion-Beam Therapy Center (HIT), Heidelberg, Germany; c German Cancer Consortium (DKTK) Core-Center Heidelberg, German Cancer Research Center (DKFZ), Heidelberg, Germany; d Clinical Cooperation Unit Radiation Oncology, Heidelberg Institute of Radiation Oncology (HIRO), National Center for Radiation Oncology (NCRO), Heidelberg University and German Cancer Research Center (DKFZ), Heidelberg, Germany; e Heidelberg Ion-Beam Therapy Center (HIT), Department of Radiation Oncology, Heidelberg University Hospital, Heidelberg, Germany; f Faculty of Physics and Astronomy, Heidelberg University, Germany; g National Centre of Oncological Hadrontherapy (CNAO), Medical Physics, Pavia, Italy; h National Center for Tumor Diseases (NCT), Heidelberg, Germany

## Abstract

**Purpose:**

To evaluate the clinical potential of spot-scanning hadron arc (SHArc) therapy with a heavy-ion gantry.

**Methods and Materials:**

A series of in silico studies was conducted via treatment plan optimization in FRoG and the RayStation TPS to compare SHArc therapy against reference plans using conventional techniques with single, parallel-opposed, and 3-field configurations for 3 clinical particle beams (protons [p], helium [^4^He], and carbon [^12^C] ions). Tests were performed on water-equivalent cylindrical phantoms for simple targets and clinical-like scenarios with an organ-at-risk in proximity of the target. Effective dose and dose-averaged linear energy transfer (LET_D_) distributions for SHArc were evaluated against conventional planning techniques applying the modified microdosimetric kinetic model for considering bio-effect with (α/β)_x_ = 2 Gy. A model for hypoxia-induced tumor radio-resistance was developed for particle therapy with dependence on oxygen concentration and particle species/energy (Z_eff_/β)^2^ to investigate the impact on effective dose.

**Results:**

SHArc plans exhibited similar target coverage with unique treatment attributes and distributions compared with conventional planning, with carbon ions demonstrating the greatest potential for tumor control and normal tissue sparing among the arc techniques. All SHArc plans exhibited a low-dose bath outside the target volume with a reduced maximum dose in normal tissues compared with single, parallel-opposed, and 3-field configuration plans. Moreover, favorable LET_D_ distributions were made possible using the SHArc approach, with maximum LET_D_ in the *r* = 5 mm tumor core (~8 keVμm^-1^, ~30 keVμm^-1^, and ~150 keVμm^-1^ for *p,*^4^He, and ^12^C ions, respectively) and reductions of high-LET regions in normal tissues and organs-at-risk compared with static treatment beam delivery.

**Conclusion:**

SHArc therapy offers potential treatment benefits such as increased normal tissue sparing. Without explicit consideration of oxygen concentration during treatment planning and optimization, SHArc-C may mitigate tumor hypoxia-induced loss of efficacy. Findings justify further development of robust SHArc treatment planning toward potential clinical translation.

## Introduction

The growing prevalence of proton gantry systems using raster-scanning technology is facilitating the development and widespread use of sophisticated approaches to targeting and treating deep-seated tumors, such as intensity modulated particle therapy (IMPT).^1^^,^^2^ Although the root concepts date back to the 1990s,^3^ clinical interest in proton arc techniques is on the rise, offering high-dose reductions in adjacent healthy tissues in the form of a low-dose bath compared with multifield IMPT. Initiated decades prior, study of proton arc techniques has demonstrated unique advantages over fixed-beam treatment delivery despite use of more elemental systems, that is, passive scattering compared with present-day state-of-the-art systems.^4^ Recently, significant progress has been made in establishing robust and deliverable arc treatments with proton beams,^5^, ^6^, ^7^, ^8^, ^9^ demonstrating potential for improved treatment efficacy in several site-specific studies. These works have consequently led to partnerships with industry to increase accessibility of arc treatment planning and delivery with new-age gantry systems.^6^ Moreover, efficient algorithms and delivery techniques like spot-scanning proton arc (SPArc) and proton arc therapy (PAT) for minimizing energy layer selection and enhancing linear energy transfer (LET) midtarget have been explored.^10^, ^11^, ^12^, ^13^ However, works have yet to investigate clinical viability of arc techniques beyond proton beams and in the context of more clinically relevant endpoints. Several key parameters related to biophysical implications of arc therapy with particle beams remain unknown or undefined.

To that end, a major shortcoming of conventional radiation therapy is lack of patient specificity in treatment planning and integration of both measurable and immeasurable characteristics of individual cells and the tumor microenvironment.^14^ Tumor hypoxia is one of the main radiation therapy resistance indications linked to poor prognosis and is not explicitly considered during treatment design.^15^^,^^16^ Spatial distributions of tumor hypoxia can vary widely between indications and even in the form of multiple diffuse regions; however, anatomic treatment sites of interest (eg, with head and neck [H&N]/non-small cell lung cancers) often present a single confluent area (tumor core) of hypoxia.^17^^,^^18^ High-LET radiation damage inherent to heavy-ion therapy shows promise in combating such resistances to treatment, especially near and within the Bragg peak (BP) end-of-range.^19^ Mainstream particle therapy treatment fields, however, involve static beam delivery at specified angles and spot selection that overlap and effectively mix entrance channel (low-LET) and BP (high-LET) dose deposition to generate the planned target dose distribution. In turn, stark LET gradients, and hence relative biological effectiveness (RBE) uncertainty, are produced at the distal edge where tumor models do not predict substantial hypoxia and where critical structures or organs at risk may reside.^20^ The innovation of novel delivery approaches to converge high-LET components toward the tumor center away from target/normal tissue boundaries would be largely beneficial for both enhancing tumor control and reducing likelihood of toxicity. Considering enhanced targeting and bio-effect properties of heavier ions, we hypothesize that arc delivery techniques using helium or carbon ions may afford more physically and biologically favorable treatment characteristics, such as increased and reduced high-LET components in hypoxic tumor regions and normal tissues, respectively.

**Figure 1:**
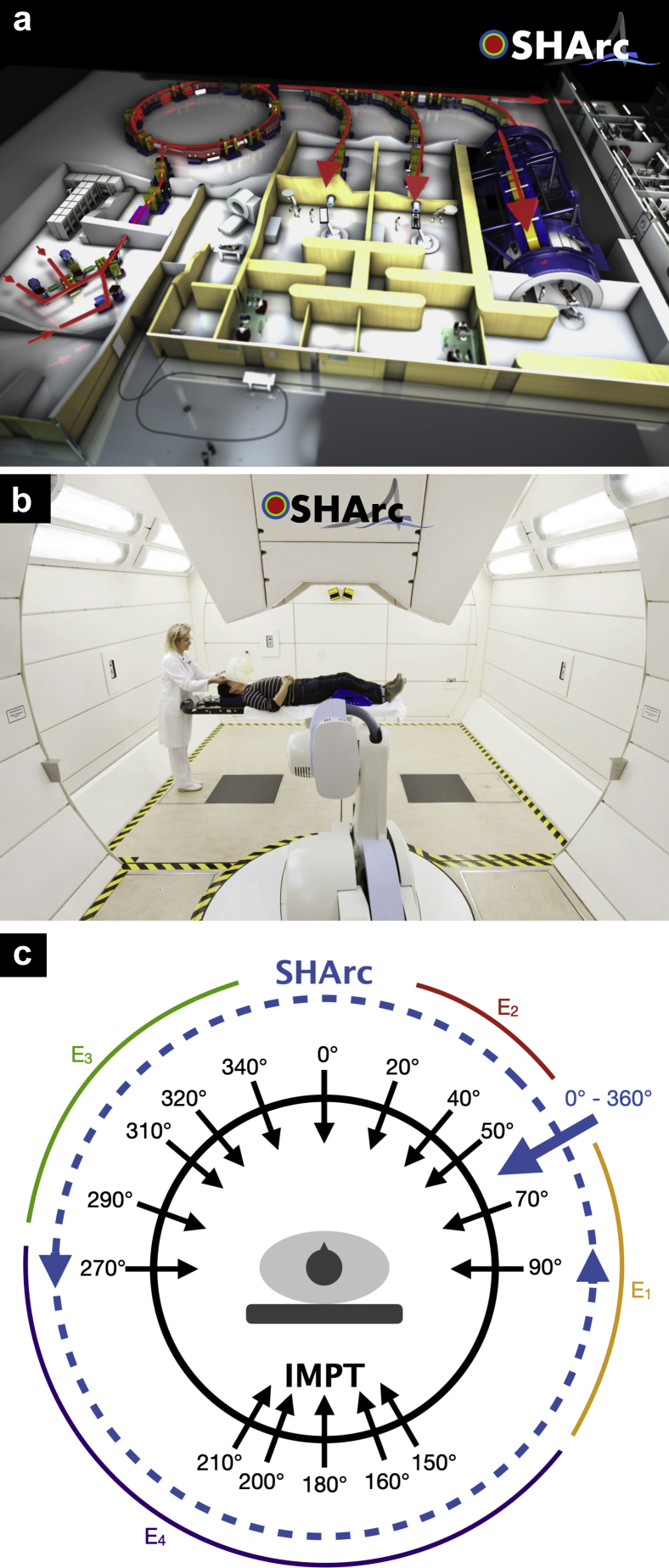
(a) 2020 facility schematic and (b) the first heavy-ion gantry system located at the Heidelberg Ion-beam Therapy Center (HIT), where spot-scanning hadron arc (SHArc) is under development. The schematic depicts multiple ion sources, linear accelerator (LINAC), synchrotron, high energy beam transport line (HEBT), fixed-beam treatment room, gantry system/HEBT, and gantry nozzle/delivery room. (c) Proof-of-concept diagram for SHArc, the first arc treatment delivery technique using a synchrotron-based delivery system for heavy ions, distinguishes conventional intensity modulated particle therapy (IMPT) treatment delivery (static approach with fixed beam angles) and arc delivery with select energies (eg, potential approach for subarc of energies E_1_, E_2_, E_3_ and E_4_). Black arrows highlight commissioned gantry angles commonly used at our clinic.

In this work, we introduce spot-scanning hadron arc (SHArc) therapy using proton (p), helium (^4^He) and carbon (^12^C) ion beams, the 3 clinical ions available at the Heidelberg Ion-beam Therapy Center (HIT).^21^ Home to the first heavy-ion gantry system (Fig 1),^22^ HIT is uniquely positioned to treat radio-resistant diseases with innovative approaches to therapy. Presently, clinical potential of particle beam arc techniques beyond proton therapy is absent in the literature. Here, we apply arc techniques to light and heavy ions and survey in silico the potential to improve tumor conformity, reduce organ at risk (OAR) dose, and enhance target LET distributions. Through characterization of dosimetric and biophysical features of SHArc therapy and development of a phenomenological model for tumor hypoxia, a series of tests investigates the merit of arc delivery in particle therapy at large.

Principle efforts in research and development at HIT focus on advanced treatment development and translation, most notably the world’s first clinical program for raster-scanning helium ion beam therapy, scheduled for mid-2021, as well as multi-ion therapy (MIT). Here, the SHArc therapy concept was preliminarily tested with the 3 clinical particle beams, outlining observed treatment characteristics using a single ion species.

## Methods and Materials

### SHArc treatment design, planning, and dose/dose-averaged LET calculation

Treatment plan design and computation of dose and dose-averaged LET (LET_D_) for SHArc therapy were conducted using RayStation Version 10A and the PRECISE TPS,^23^ based on the graphics processing unit (GPU) accelerated dose engine FRoG, respectively.^24^, ^25^, ^26^ Cylindrical water phantoms were generated (H = 200 mm, r = 100 mm) with a cylindrical target situated at isocenter (H = 60 mm, r = 30 mm). As reference, conventional treatment plans were optimized using 1 (90°), 2 (0°/180°), and 3 (0°/90°/180°) beam configurations for p, ^4^He, and ^12^C ions. Beam settings for foci and implementation of a ripple filter followed clinical procedure. Two complete sets of IMPT and SHArc optimizations were performed for the following scenarios:

Case A: Simple phantom study with target and normal tissue optimization criteria
Case B: Clinical-like setting with planning target volume (PTV), OAR, and normal tissue optimization criteria

For SHArc, plans exploited the full gantry rotation window (360°) with 2° angular sampling. For feasibility of arc delivery with the synchrotron at our facility (ø energy switching), a monoenergetic beam was determined by selecting the BP where R_80_ ≈ ½ cylinder radius: 118.14 MeV/u for p*,* 118.51 MeV/u for ^4^He, and 218.52 MeV/u for ^12^C. Lateral spot separation was set to 4.8 mm, 2.4 mm, and 2.4 mm for p*,* ^4^He, and ^12^C ions, respectively. Effective dose was computed using the modified microdosimetric kinetic model (mMKM) for saturation-corrected dose-mean specific energy of the domain delivered in a single event $\left({\text{z}}_{1\text{D}}^{\text{∗}}\right)$^27^^,^^28^ with best fit values R_d_ = 0.3 μm and R_n_ = 3.6 μm, obtained through fitting in vitro data for p and ^4^He ion beams.^29^^,^^30^ The mMKM applied in this work has been shown to successfully predict RBE in vitro and in vivo for carbon ion therapy.^31^ Reference photon tissue fractionation parameter (α/β)_x_ = 2 Gy was set (α_x_ = 0.05 Gy^-1^ and β_x_ = 0.025 Gy^-2^). Further information regarding effective dose modeling for ion-beam therapy is provided in the appendix of the Supplementary Materials*.*

For all cases, prescription dose was set to 3 GyRBE with target coverage objective functions of 106% and 97% upper and lower limits, respectively, and a lateral dose fall-off constraint of 0.25 GyRBE within 5 mm of the target boundary. For the clinical-like scenario with a cylindrical OAR (case B), a maximum OAR dose objective of 0.5 GyRBE was set. Resultant D_RBE_ and LET_D_ predictions were optimized and calculated for each case, respectively. For consistency between the ion species while remaining in the scope of proof-of-concept, optimizations for target coverage were performed using the entire set of selected beam angles and spot positions. Before optimization, initial inputs for spot selection covered the entirety of the target volume in the beam’s eye view for each beam angle. During optimization, while achieving defined objectives, spot number minimization took place and clinical thresholds for minimum fluence per spot were applied with 5.8 × 10^5^, 1.3 × 10^5^, and 1.5 × 10^4^ for p*,* ^4^He, and ^12^C, respectively. Identical optimization and calculation settings were applied for the IMPT single, parallel-opposed, and 3 field configurations (1F/2F/3F) plans for p*,* ^4^He, and ^12^C ions.

### Modeling hypoxia/oxygen enhancement ratio (OER)

Cells under hypoxic conditions exhibit increased radio-resistance, and consequently, disease sites containing hypoxic regions are known to lower probability of local control using radiation therapy.^32^^,^^33^ Higher LET particle beams such as ^12^C and ^16^O ions have been suggested^34^, ^35^, ^36^ as a means to improve clinical outcome in treating hypoxic tumors due to their ability to reduce the OER. The OER is used to quantify the cell survival dependence on the oxygenation status and is typically defined as the ratio between iso-effective doses in a hypoxic and a normoxic environment.^33^ A model to predict OER based on oxygen concentration (pO_2_) for a particular ion species was developed to evaluate potential improvements in overcoming tumor hypoxia radio-resistance for IMPT versus SHArc treatments. The model employs a phenomenological approach to fitting collected in vitro data from the literature (Fig E1). As opposed to previously published phenomenological/mechanistic models to describe the OER decreasing as a function of LET, the model presented here considers the mixed-radiation field spectra in terms of the particle spectra (ie, primary and secondary fragments) as a function of energy and depth in water.^37^ The model was incorporated into FRoG for effective dose calculation in hypoxic tumors.

#### Experimental data from literature

To phenomenologically model hypoxia-induced radio-resistance, normoxia and hypoxia cell survival data were extracted from the literature for p*,* ^4^He, and ^12^C ions. Linear quadratic parameters were derived by fitting the data with an in-house tool based on the CERN ROOT framework (http://root.cern.ch) and the MINUIT minimization package (Brun and Rademakers 1997). V79 was the most frequently investigated cell line in the collected publications.^38^ Most works in the literature described the radiation quality of p*,* ^4^He, and ^12^C ion beams in terms of LET. It is important to note, however, that there is an intrinsic uncertainty within the collected LET values. Specifically, LET was not always unequivocally calculated; some publications used either dose-averaged LET or track-averaged LET, whereas others did not specify. Beam energy information (when unreported) was obtained by interpolating the LET-energy database in water used in FRoG. Further details regarding the modeled data are provided in the appendix of the Supplementary Materials.

#### Modeling approach

The model is comprised of various parameterizations under the formalism of a hypoxia reduction factor (HRF) to incorporate particle, energy, and pO_2_ dependencies into RBE prediction. The model has been generalized for all particle species/energy as a function of (Z_eff_/β)^2^ and pO_2_.^39^ Z_eff_ is the effective charge, and β = v/c (relative particle velocity normalized by the speed of light). With photons, the $HRF_{ph}^{O_{2}}$ can be estimated from the parameterization:

(1) $$HRF_{ph}^{O_{2}}\left(\left[O_{2}\right]\right)=\frac{m\cdot K+\left[O_{2}\right]}{K+\left[O_{2}\right]}$$

which was introduced in previous works,^40^^,^^41^ proposed in reference^42^ and inspired by the initial works of the authors in reference^43^. Fitting this parameterization to data available in the literature, values *m* = 2.94 and *K* = 0.129% were obtained. When both hypoxic and normoxic survival data for a specific cell line were available at 2 different O_2_ levels, *m* and *K* in equation 1 were obtained by fitting the data. For higher LET particles, one must include LET/beam energy dependency into $HRF_{ion}^{O_{2}}$. Wenzl and Wilkens^44^ developed an OER model with parameters dependent on LET and pO_2_ using experimental data from several particle species. Dahle and collaborators^45^ developed an LET-based model for protons assuming a survival fraction of 10%. Scifoni et al (2013)^35^ and Tinganelli et al (2015)^36^ described a biological dose model dependent on the OER, intended mainly for heavier ions like ^12^C and ^16^O. More mechanistic approaches have been published in the literature.^46^ Stewart et al^39^ in particular used ${\left(Z_{eff}/\beta \right)}^{2}$; that is, the ratio of the square of the effective charge and the square of the particle’s speed relative to the speed of light as the preferred indicator of radiation quality. In this work we developed an approach to describe HRF based on ${\left(Z_{eff}/\beta \right)}^{2}$, specifically handling the individual contribution of each particle species within the mixed radiation field spectra. The effective charge is calculated according to Barkas et al.^47^ The experimental $HRF_{ion}^{O_{2}}$ has been calculated as described in literature^44^ within the LQ framework (denoting ${\left(Z_{eff}/\beta \right)}^{2}$ as RQE, representing the radiation quality energy dependency):

(2) $$HRF_{ion}^{O_{2}}\;\left(RQE,\;pO_{2}\right)=\frac{\sqrt{\alpha^{2}\left(RQE,pO_{2}\right)-4\beta \left(RQE,pO_{2}\right)\cdot ln\left(S\right)}-\alpha \left(RQE,pO_{2}\right)}{\sqrt{\alpha^{2}\left(RQE\right)-4\beta \left(RQE\right)\cdot ln\left(S\right)}-\alpha \left(RQE\right)}\cdot \frac{\beta \left(RQE\right)}{\beta \left(RQE,pO_{2}\right)}$$

$\alpha \left(RQE,pO_{2}\right)$, $\beta \left(RQE,pO_{2}\right)$ and α(RQE), β(RQE) values represent the LQ parameters in hypoxic (pO_2_ level) and in normoxic conditions, respectively, for survival *S*. To evaluate $HRF_{ion}^{pO_{2}}$ in clinically relevant conditions, a typical fractionated dose level for proton therapy (~2 Gy) was assumed. In line with the work of Carlson et al (2006),^42^ Mairani et al (2013),^40^ and Scifoni et al (2013),^35^ we have assumed that in a first approximation $HRF_{ion}^{O_{2}}$ is a dose modifying factor at any survival level. Then $\alpha \left(RQE,O_{2}\right)$ and $\beta \left(RQE,O_{2}\right)$ can be obtained from the normoxic values by references 32 and 39. In short, the α_ion_ and β_ion_ (calculated by the mMKM version outlined in the appendix of the Supplementary Materials) are normalized by the HRF, given as

(3.a) $$\alpha_{ion}^{pO_{2}}\left(\text{Γ},pO_{2}\right)=\frac{\alpha_{ion}\left(\text{Γ}\right)}{HRF_{ion}^{pO_{2}}\left(\text{Γ},pO_{2}\right)}$$

and

(3.b) $${\text{β}}_{ion}^{pO_{2}}\left(\text{Γ},pO_{2}\right)=\frac{{\text{β}}_{ion}\left(\text{Γ}\right)}{HRF_{ion}^{pO_{2}}{\left(\text{Γ},pO_{2}\right)}^{2}}$$

where Γ = (Z_eff_/β)^2^. Trends for $\alpha_{ion}^{pO_{2}}$ and ${\text{β}}_{ion}^{pO_{2}}$ dependency as a function of beam energy with various pO_2_ levels are provided in the appendix of the Supplementary Materials*.* The radiation quality and LET/energy dependence for $HRF_{ion}^{O_{2}}$ was parametrized following the work of Stewart et al^39^ in terms of ${\left(Z_{eff}/\beta \right)}^{2}$:

(4) $$HRF_{ion}^{pO_{2}}\left(RQE\right)=\frac{a\cdot HRF_{ph}^{pO_{2}}\left(\left[pO_{2}\right]\right)+RQE^{\gamma }}{a+RQE^{\gamma }}$$

**Figure 2:**
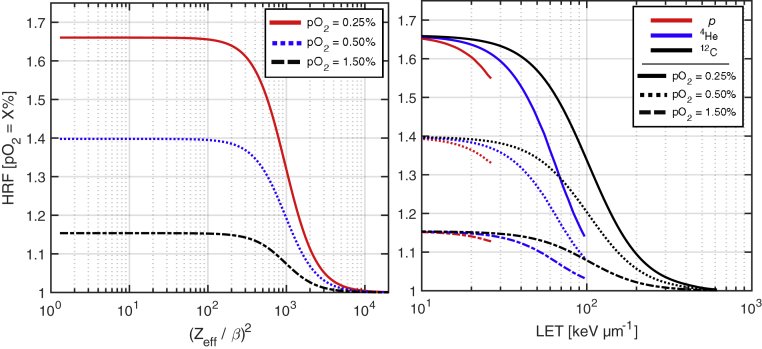
Hypoxia reduction factor (HRF) as a function of ${\left(Z_{eff}/\beta \right)}^{2}$for various pO_2_ levels (left). HRF-linear energy transfer (LET) trends are displayed for p, ^4^He, and ^12^C. Clinically relevant LET ranges are presented for each ion.

In equation (4), $HRF_{ph}^{O_{2}}$ represents the limit of $HRF_{ion}^{O_{2}}$ with RQE toward 0, a = 2.988 × 10^6^, and γ = 2.169 (set to reproduce the steeper fall-off toward higher RQE). Figure 2 depicts modeled HRF dependencies as a function of ${\left(Z_{eff}/\beta \right)}^{2}$for 3 relevant pO_2_ levels as well as within the clincally relevant LET range for p, ^4^He, and ^12^C ions.

### Model application and analysis

The model was integrated into FRoG for calculation in hypoxic tumors for p*,* ^4^He, and ^12^C ions. To perform clinical OER calculations, a mixed radiation field in terms of particle species and kinetic energy must be readily handled, for example, as explained in Kopp et al, 2020^37^ by generating particle spectra with Monte Carlo simulation for FRoG. In this work, we applied the mMKM-based biological dose framework for optimization and calculation of biological databases with Monte Carlo simulation (see the appendix of the Supplementary Materials).

The HRF model was employed to the clinical-like scenario (case B) applying pO_2_ gradients to the PTV to simulate in vivo conditions, with levels ranging from 5% to 0.25% from the outer ring to the inner tumor core, logarithmically spaced in 9 intervals. Outside the target volume pO_2_ = 21% (normoxic condition) was applied. Forward calculations were performed to determine the influence of hypoxia-related radio-resistance on D_RBE_ distributions for all SHArc treatments and 2F conventional plans in the clinical-like scenario (case B). During evaluation and assessment of treatment plan optimization, the ratio between normoxic effective dose (ie, D_RBE_) and hypoxic effective dose (D_OER,RBE_) was defined as follows:

(5) $$\Delta_{\text{OER},\text{RBE}}\left({\text{pO}}_{2}\right)=\frac{{\text{D}}_{\text{RBE}}}{{\text{D}}_{\text{OER},\text{RBE}}}$$

A detailed description of D_OER,RBE_ calculation with mMKM is provided in the appendix of the Supplementary Materials. Furthermore, to investigate potential effect on treatment efficacy, tumor control probability (TCP) was calculated, defined as

(6) $$TCP\left(n\right)=\prod_{i=\;1}^{N}e^{-S_{i}^{n}v_{i}p_{i}}\;$$

using the survival (S) prediction based on the applied tumor hypoxia conditions for *n* fractions and *N* voxels in the PTV, with voxel (i) size *v* = 1 mm^3^ and *p* = 10^4^ cells/mm^3^ as outlined in prior studies.^36^

## Results

**Figure 3:**
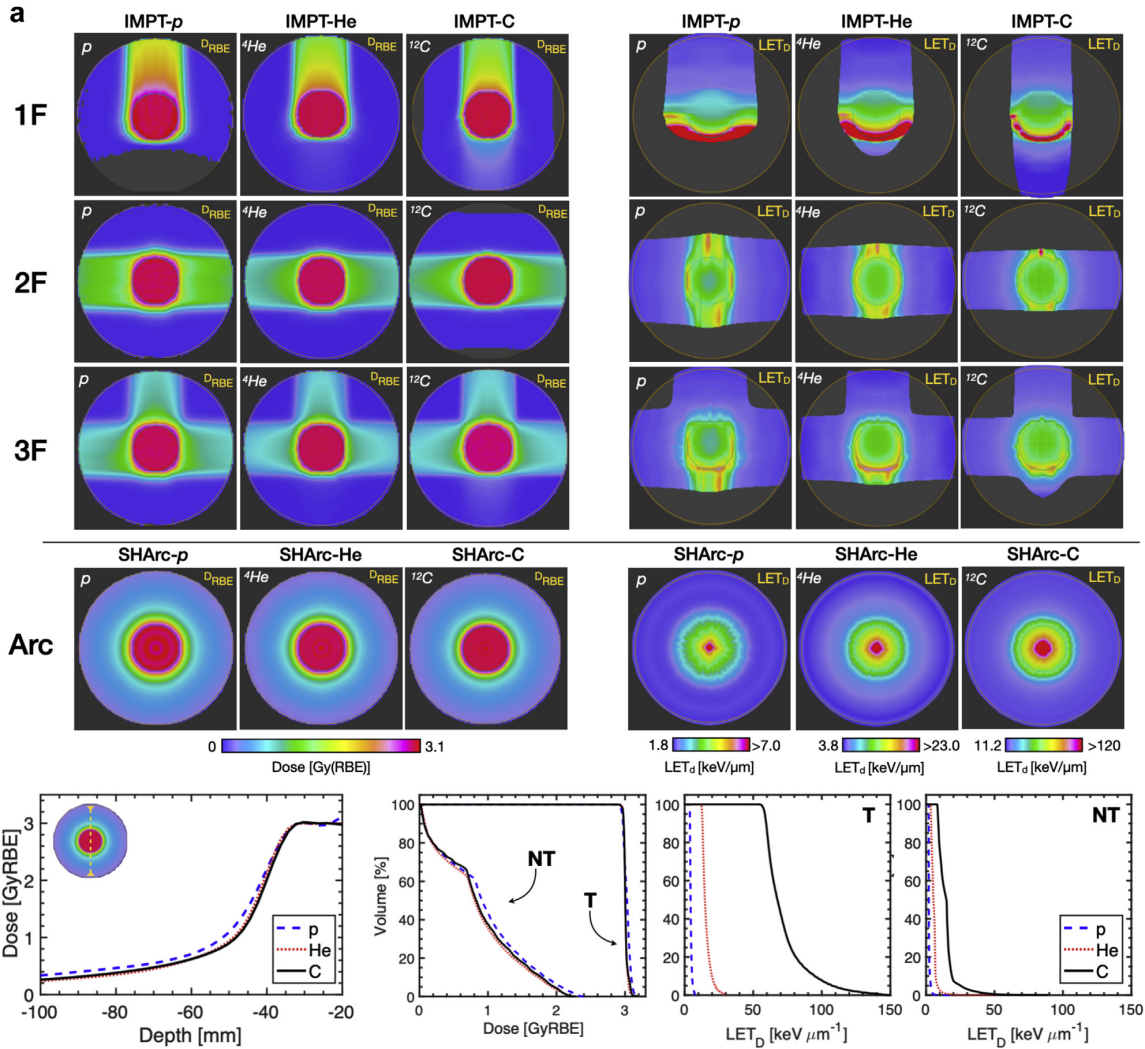

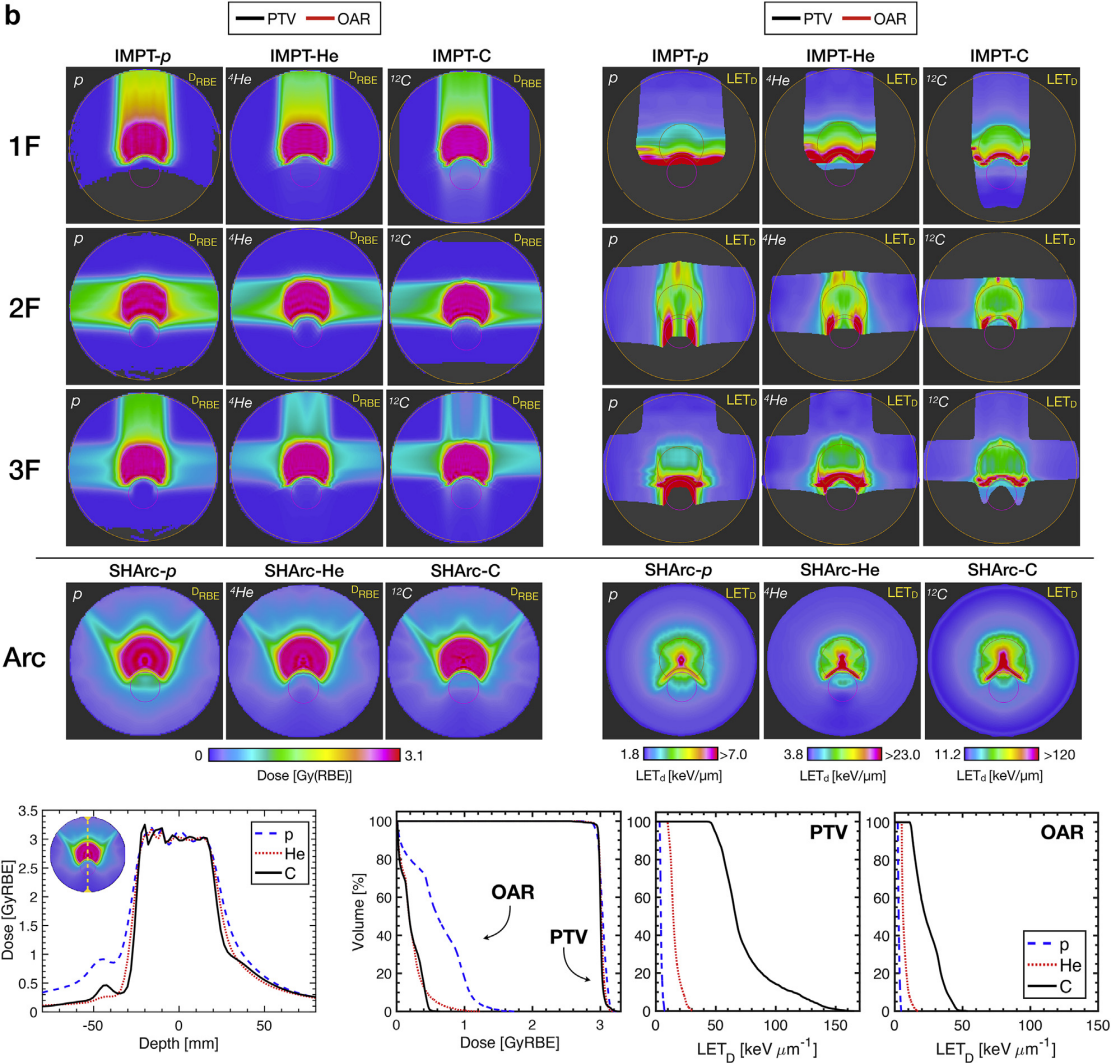

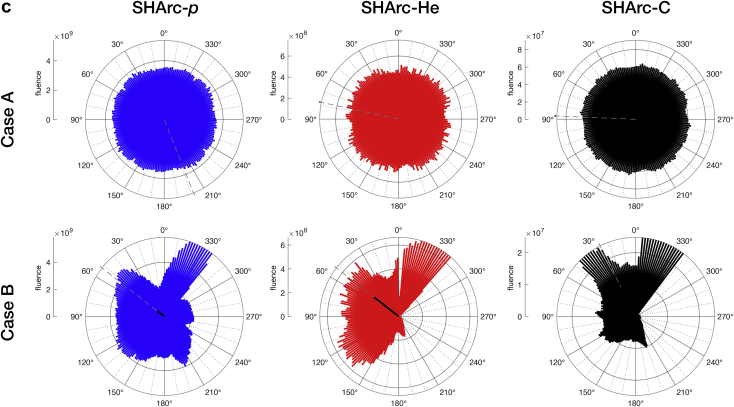
D_RBE_ and dose-averaged linear energy transfer (LET_D_) maps for intensity modulated particle therapy (IMPT) versus spot-scanning hadron arc (SHArc). (a) Case A: optimization with target (T) and normal tissue (NT) constraints. (b) Case B: clinical-like scenario with planning target volume (PTV)/organs at risk (OAR) optimization. Both cases were conducted using 3 clinical ion beams (p*,*^4^He, and ^12^C ions). Line profiles, dose volume histogram (DVH), and dose-averaged linear energy transfer volume histogram (LET_D_VH) are provided for intercomparison of SHArc plans (bottom panels). (c) Angular-fluence maps for SHArc-p*,* SHArc-He, and SHArc-C plans in cases A (top) and B (bottom).

For both optimization cases A and B, 1F/2F/3F conventional and SHArc treatment optimizations were successfully performed following the clinical constraints, reaching an average target dose of ~3 GyRBE. Dose, LET_D_, and angular-fluence maps are presented in Figure 3. For case A (Fig 3a), SHArc plans yielded comparable target coverage accompanied by a low-dose bath surrounding the target volume compared with the conventional treatments. Among the SHArc treatments, helium and carbon ions exhibited the greatest normal tissue sparing, as demonstrated in the line profiles and dose volume histogram (DVH), with entrance dose values increasing from ~0.3 GyRBE to ~0.5 GyRBE from the entrance to 10 mm radially away from the target boundary.

For the 1F/2F/3F treatments, maximum LET_D_ (LET_D_,_max_) was located at the distal-end/outside of the target, while for SHArc treatments, LET_D_,_max_ was located within the central core of the target volume (~8 keV･μm^-1^, ~30 keV･μm^-1^, and ~150 keV･μm^-1^ for p*,* ^4^He, and ^12^C ions, respectively). LET_D_,_max_ in the normal tissues were substantially reduced for SHArc treatments, up to ~73% compared with the 1F treatments. For the SHArc treatments, ^12^C ions exhibited the sharpest penumbra and lowest D_RBE_ outside the target.

For the clinical-like optimization scenario with OAR consideration (case B), tumor coverage was comparable between SHArc and IMPT planning; however, great variations in LET_D_ distributions were observed. Particularly for the 2F/3F IMPT treatments, LET_D_ at the distal edge and beyond the PTV within the OAR increase substantially compared to SHArc on the order of 60% from ~6 keV･μm^-1^ to ~15 keV･μm^-1^ for protons*,* ~16 keV･μm^-1^ to 33 keV･μm^-1^ for helium ions, and ~78 keV･μm^-1^ to ~135 keV･μm^-1^ for carbon ions. In contrast, IMPT-C, SHArc-p, SHArc-He, and SHArc-C treatments provided better sparing in terms of high-LET_D_ components in the OAR. However, unlike SHArc delivery, IMPT-C pushed LET_D,max_ in normal tissues outside of the target, and exhibited a significantly lower midtarget LET_D_ compared with SHArc-C.

Between the SHArc plans, helium and carbon ions exhibited superior performance in balancing OAR sparing and target coverage, with a slight advantage when using helium as a result of overlapping carbon ion fragmentation tails, visualized as a “bump” of elevated dose in the line profiles (Fig 3b). A more pronounced effect was observed for SHArc-p*,* primarily due to increased beam spread end-of-range, sizeable low-dose halo (non-Gaussian beam shape), and nonzero particle fluence for beam angles with OAR in the beam’s eye view (210°-300° in SHArc-p angular-fluence map, Fig 3c). For the given inputs and constraints, SHArc-p optimization could not be further improved for reducing maximum dose (D_max_) within the OAR without greatly sacrificing target coverage.

**Figure 4:**
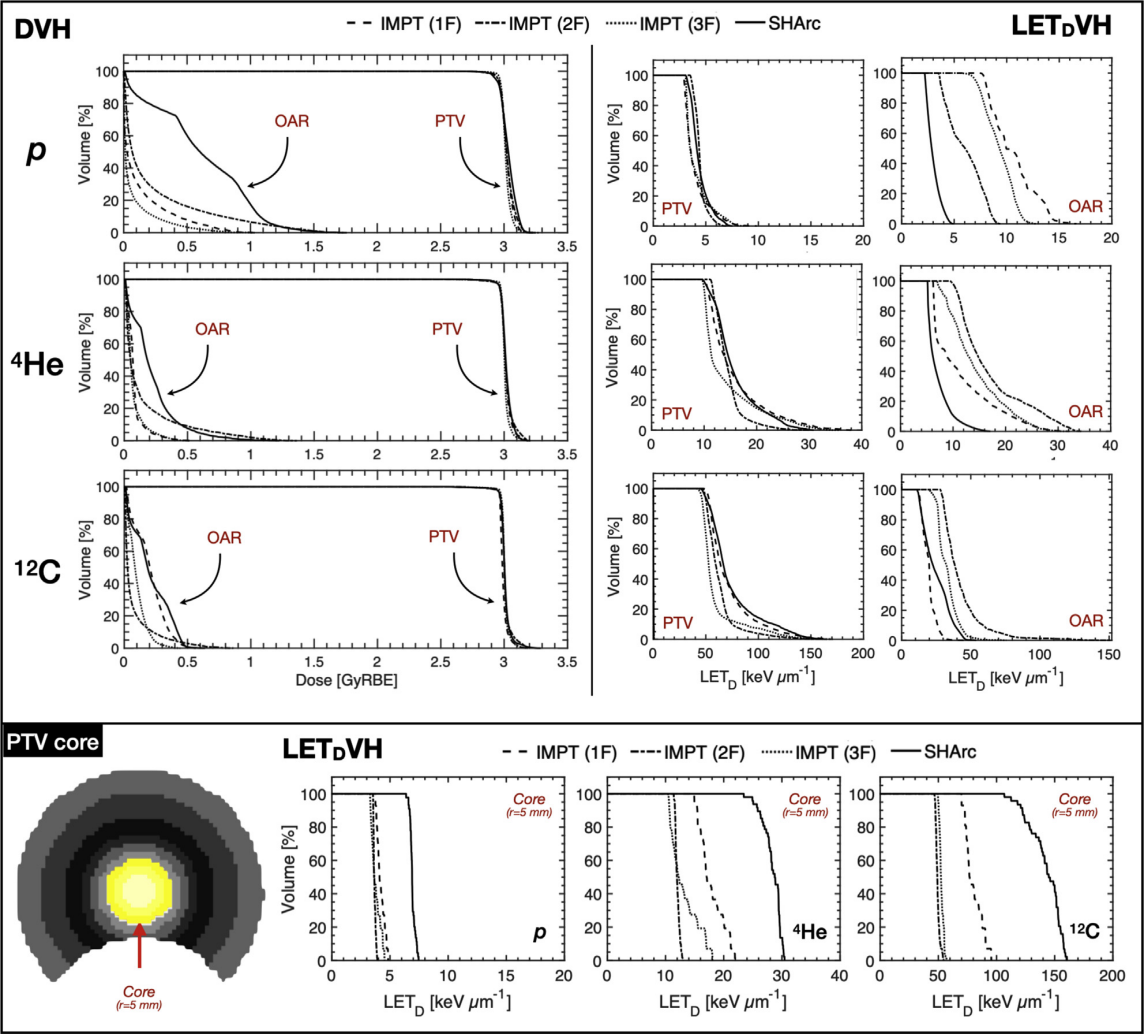
Dose volume histogram (DVH) (top left) and dose-averaged linear energy transfer volume histogram (LET_D_VH) (top right) for intensity modulated particle therapy (IMPT) (1F/2F/3F) versus spot-scanning hadron arc (SHArc) using p*,*^4^He, and ^12^C ions for case B with planning target volume (PTV), organs at risk (OAR), and normal tissue criteria/constraints. LET_D_VH for *r* = 5 mm PTV core is presented (bottom) with graphic highlighting considered volume. LET axes are scaled for relative comparison of each particle species.

For case B, DVH and dose-averaged linear energy transfer volume histogram (LET_D_VH) are provided for the PTV and OAR (Fig 4). For similar coverage compared with conventional planning, SHArc-C could effectively meet clinical OAR constraints with D_max,OAR_ < 0.5 GyRBE. D_2%,OAR_ values were 0.78 GyRBE, 0.49 GyRBE, 0.35 GyRBE for IMPT-C 1F, 2F, and 3F, respectively, while D_2%,OAR_ for SHArc-C was 0.47 GyRBE. SHArc-He performed similarly with D_2%,OAR_ less than of IMPT-He 2F, but more than 46% higher than IMPT-He 1F/3F configurations. SHArc-p exhibited the least OAR dose sparing.

Examination of LET_D_ distribution within the PTV revealed unique features for SHArc compared with IMPT planning, particularly for the *r* = 5 mm core (LET_D_VH, Fig 4). All 3 SHArc plans delivered substantially higher LET_D_ to the central core of the PTV, with LET_D_ delivered to 50% of the volume (LET_D,50_) of ~7 keV･μm^-1^, ~28 keV･μm^-1^, and ~150 keV･μm^-1^ for p*,* ^4^He, and ^12^C ions, respectively. On average, SHArc LET_D,50_ values were 93%, 121%, and 142% higher than IMPT in the central core for p*,* ^4^He, and ^12^C ions, respectively.

**Figure 5:**
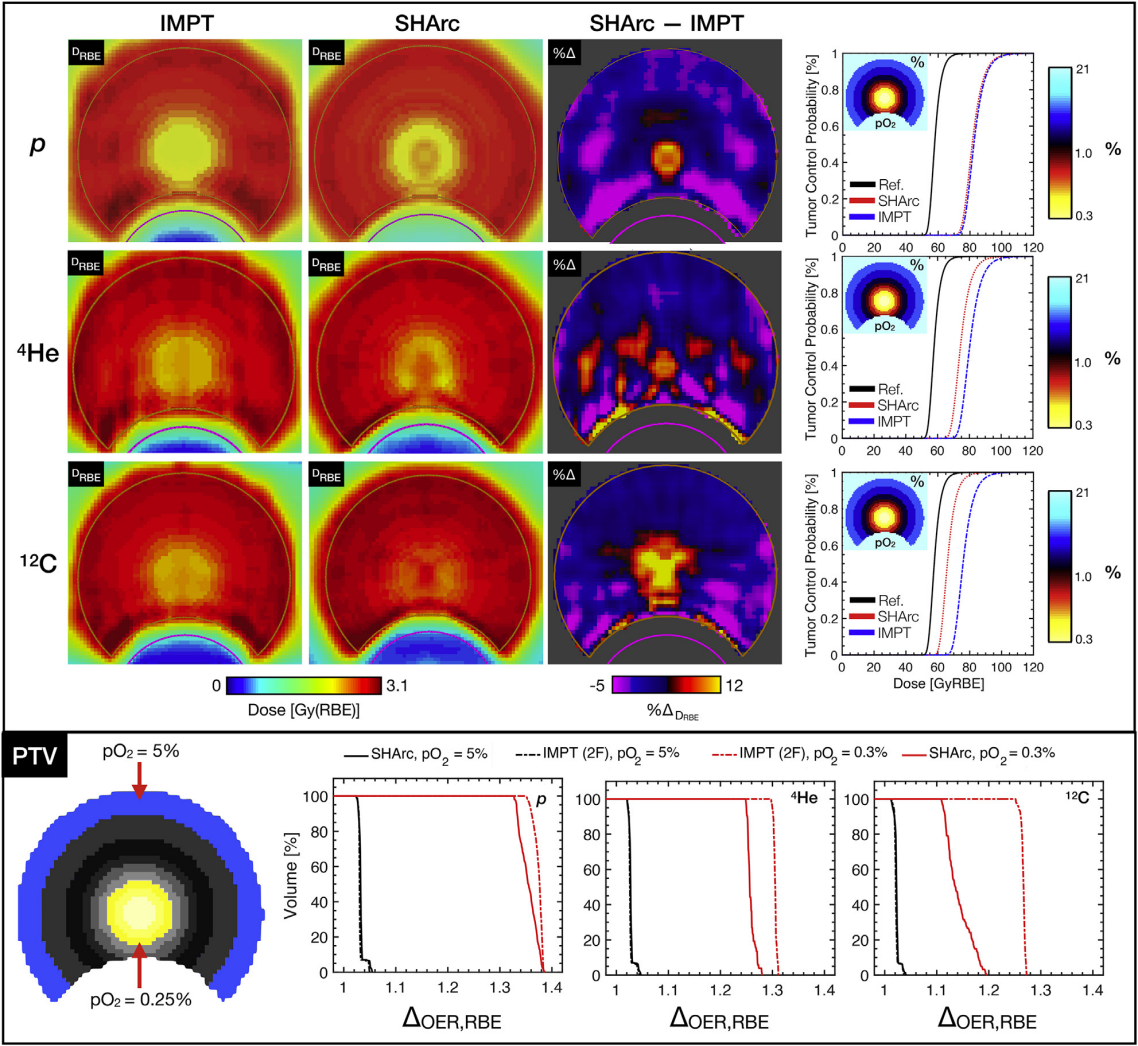
Assessing radio-resistance induced by tumor hypoxia (planning target volume [PTV]) for particle therapy with p*,*^4^He, and ^12^C ions. D_RBE_ maps for spot-scanning hadron arc (SHArc) therapy, intensity modulated particle therapy (IMPT), and subtractions given in terms of percent difference from the prescription dose for case B applying the phenomenological hypoxia reduction factor (HRF) model. pO_2_ maps are presented alongside tumor control probability (TCP) calculations for SHArc and IMPT deliveries under set conditions of tumor hypoxia compared with reference normoxic conditions (Ref.). Δ_OER,RBE_ plots are provided (bottom) for IMPT-2F versus SHArc plans for the outer PTV ring (pO_2_ = 5%) and inner PTV core (pO_2_ = 0.25%) for p*,*^4^He, and ^12^C.

Effect of tumor hypoxia on target coverage is presented (Fig 5) with forward calculation D_RBE_ maps for SHArc and IMPT treatments computed via HRF model integration within FRoG. The outer periphery of the tumor (5% ≤ pO_2_ ≤ 1%) remained relatively stable compared with the reference D_RBE_ plan (ie, normoxia) with variation on the order of 2% to 6% for all ions. For the inner PTV core *r* < 5 mm, variations in D_RBE_ from the reference plan were substantially higher particularly for protons (%Δ_D,max_ ≈ 28% for IMPT-2F and SHArc). SHArc-C exhibited the greatest propensity to overcome hypoxia-related radio-resistance compared with IMPT-2F, with %Δ_D,max_ between SHArc and IMPT of ~15% in the *r* = 5 mm PTV core. With respect to shifts in D_RBE_ with tumor control probability at 50% (TCP_50_), SHArc-C and IMPT-C increased by 7.4 GyRBE and 18.3 GyRBE (Fig 5). Changes in TCP_50_ for p and ^4^He treatments compared with reference were more substantial, with SHArc-He exhibiting slight improvements (+ 5.1 GyRBE) compared with IMPT-He. Differences in TCP_50_ between IMPT-p and SHArc-p were not significant. Δ_OER,RBE_ volume histograms (Δ_OER_VH) are additionally provided (Fig 4, bottom panel) for the outer periphery (pO_2_ = 5%) and central core (pO_2_ = 0.25%). Δ_OER,50_ for pO_2_ = 5% in the target rim ranged between 3% for carbon ions and 6% for protons. As for the inner core (*r* = 5 mm) where %Δ_D_ values are observed, average %Δ_OER_ values between IMPT and SHArc were as follows: 2.1% for p*,* 4.7% for ^4^He, and 14.1% for ^12^C.

## Discussion

At HIT, appraisal of novel treatment modalities such as SHArc is underway, beginning with theoretical studies to investigate potential clinical gains and feasibility. The findings in this work demonstrate that, in theory, arc delivery with light and heavy ions like SHArc presents numerous treatment advantages compared with conventional static IMPT approaches with single and multifield delivery. Through comprehensive study and development of a phenomenological model for hypoxia-related radio-resistance for particle beams, we present the first preliminary survey of arc therapy delivery techniques using helium and carbon ions, highlighting unique dosimetric and bio-effect features. Clinical realization of SHArc involves acknowledging and solving several technical hurdles from planning to delivery of arguably one of the most complex particle therapy treatment scenarios — raster-scanning with live rotation of the heavy-ion gantry system. Nonetheless, the evidence here underlining the potential clinical benefits justifies further development and study of SHArc therapy.

**Figure 6:**
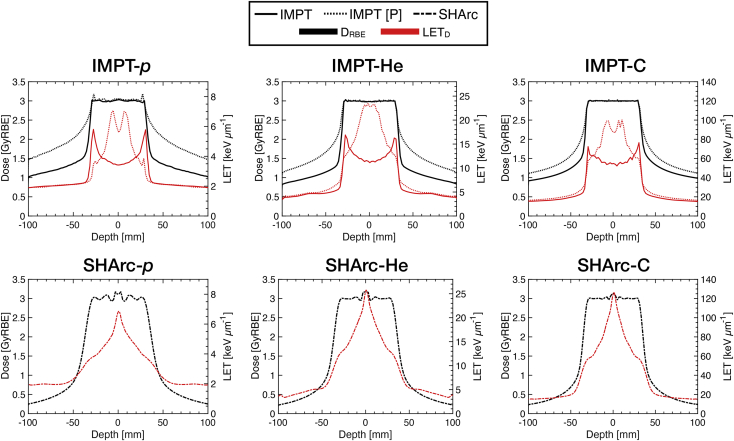
Dose and dose-averaged linear energy transfer (LET_D_) profiles for p*,*^4^He, and ^12^C using intensity modulated particle therapy (IMPT)-2F (conventional vs patch configuration [P]) plan optimization are displayed (top) with reference spot-scanning hadron arc (SHArc) plan profiles (bottom). Midtarget LET enhancement IMPT-2F[P] yields comparable values with SHArc however, IMPT-2F[P] significantly increases entrance dose, particularly for p and ^4^He on the order of ∼60%.

In summary, SHArc delivers a low-dose bath to surrounding normal tissues and a markedly enhanced targeting of high-LET (~150% higher for ^12^C) within the central regions of the tumor volume, which, in practice, cannot be achieved via conventional means without substantially increasing entrance dose. For proof-of-principle, additional IMPT-2F plans were optimized to enhance LET_D_ within the target volume (case A) to demonstrate practical procedures to increase central LET within the target volume by altering the weighting factors and intrafield iso-energy configurations, described in recent works (eg, patch optimization of two opposed downslope dose profiles [PATCH]).^48^ The PATCH technique, however, substantially increases entrance dose (Fig 6).

Regarding OAR sparing, for equal target coverage, conventional therapy using 1F/2F/3F may deliver low doses to a smaller volume than SHArc but is case/location dependent and varies with selection of beam orientation. For instance, in LET_D_VH profiles for carbon ions, SHArc and IMPT-1F are the most conservative in terms of high-LET delivery to the OAR; however, when the PTV-OAR separation is decreased from 5 mm to 3 mm, LET_D,OAR_ for IMPT-1F increases up to ~170 keV･μm^-1^. Nonetheless, the benefit of overcoming hypoxia radio-resistance by focusing high-LET components in the central target could rationalize SHArc techniques accompanied by the relatively minimal sacrifice in OAR sparing. Considering superior TCP with SHArc (Fig 5), dose escalation on radio-resistant tumors, accomplished by a second boost phase or simultaneous integrated boost, could be avoided altogether, leading to an overall reduced normal tissue toxicity and increased therapeutic ratio.

Figure 5 presents a key take-away from the analysis of hypoxia effects on target coverage. Despite the retrospective nature of the study, where pO_2_/state of tumor hypoxia was not explicitly considered during treatment planning and optimization, SHArc-C in particular demonstrated potential to reduce effects of hypoxia radio-resistance within the tumor core. HRF-LET trends in Figure 2 further support these findings, demonstrating that out of all 3 investigated ions, HRF is substantially reduced (minimal hypoxia effect) only for carbon ions with LET > 100 keV･μm^-1^. One could argue that SHArc offers a practical method for lessening the unsolicited bio-effects stemming from tumor heterogeneity and pO_2_ gradients in vivo whose measurement and incorporation are essentially absent in conventional treatment planning. Nonetheless, differences in TCP_50_ were ~7 to 8 GyRBE from SHArc-C under normoxic to hypoxic conditions for the set treatment conditions. Determination of optimal particle species/LET optimization strategies may yield further increase in tumor control. PTV core analyses (Figs 4 and 5) account for only ~10% of total PTV, providing a conservative estimate of clinical effect; however, in reality, severe hypoxia may be present in a more significant portion of the tumor (increasing ΔTCP).

To effectively overcome hypoxia-related radio-resistance at the tumor core, significantly higher LET is required than what is offered by current clinical practice with particle therapy. Figure 2 depicts HRF at various pO_2_ levels versus LET for the 3 particle species, demonstrating that for clinically relevant limits of pO_2_, high-LET particles like ^20^Ne may be the only efficient means of combating hypoxic effects in larger volume targets.^49^ Consequently, SHArc shows promise to reach necessary LET levels within the central target, otherwise unattainable with conventional techniques. Nonetheless, the presented HRF model provides insight during investigations of oxygen tension and subsequent changes in effective dose prediction for particle beams, particularly convenient for heavy ions by considering influence of the mixed-field radiation spectra on biological parameters via phenomenological modeling.

In the context of normal tissue toxicity, one must note that D_RBE_ predictions for normal tissue and OARs are expressed in terms of cell-kill RBE, and thus, to make distinct arguments regarding effect on normal tissue, bio-experimentation and measurement of more relevant toxicity-related endpoints are required. D_RBE_ prediction, particularly for carbon ion beams, exhibits substantial uncertainty on the order of 20% to 30% with model/input parameter dependencies outlined in recent studies.^31^ Additional SHArc-C optimizations were performed applying the National Institute of Radiologic Sciences (NIRS)-based definition for RBE-weighted dose with ~3 GyRBE target dose, and average D_RBE_ in normal tissues for case A were ~8% lower (data not shown). Supplementary characterizations will perform sensitivity studies and survey biological dose uncertainty for SHArc, for example, on various tissue type assumptions applied in particle therapy, specifically tissue parameter assignment (α/β)_x_ and corresponding absolute values.

During optimization, particularly for case B (Fig 2b), obtaining ideal/uniform target coverage comparable to the conventional treatments was challenging for SHArc-p*,* and consequently, a noticeable increase in OAR dose was presented (Fig 3). This setback may be due to the relatively large spot-size and secondary dose-envelope using our facility settings for proton beams; however, recent works additionally acknowledge limitations in ensuring similar coverage in proton arc planning as IMPT.^12^

In related works regarding development of novel particle therapy treatment modalities, MIT is proposed to reduce LET and bio-effect related-uncertainties in treatment outcome, generating more homogenous physical/biological distributions in the target, that is, physical dose, LET, and RBE.^31^ Merging arc techniques with MIT strategies (eg, combining ≥2 ion species and partial arc delivery, Fig 1c) may provide additional benefits and compromise for both the desired homogeneity/target distribution qualities and reductions in normal tissue doses. Moreover, hypo-fractionation treatments (>>4 GyRBE/Fx) with SHArc and/or MIT may offer ideal treatment scenarios for meeting OAR constraints while significantly reducing treatment course length.^50^^,^^51^

Despite considerable evidence that poor prognosis (ie, relapse) in both radio- and chemotherapy is linked to increased resistance to therapy in oxygen-deprived tumor cells, simply measuring and incorporating hypoxia-related effects remains a major impedance in effectively eliminating invasive solid tumors. Consequently, techniques to image and overcome radio-resistance in the tumor microenvironment, such as tumor hypoxia and heterogeneity, are of particular interest in the field of particle therapy.^52^ High-LET or hypo-fractionation treatment schemes show promise to reduce such effects as well as potential toxicity in the normal tissues at the proximal portions (entrance channel) of the patient.^53^ Recent works propose single and multi-ion kill painting^35^^,^^36^^,^^54^^,^^55^ to focus high-LET beams within the hypoxic tumor core, which in principle would rely on biologically informed optimization, that is, knowledge of pO_2_ distributions within the tumor volume. The ultra-high dose rate (FLASH) phenomena, that is, dimished severity of normal tissue toxcities at high dose level/rate, is an ongoing debate in the particle community, relying on sparse, conflicting data, unverified mechanisms of action, and experimental settings that are challenging to ensure and replicate between centers. In this context, arc techniques may provide a more practical means of boosting dose rates and in turn reducing normal tissue toxicities.^56^

A crucial element of SHArc delivery is availability of not only heavy ion accelerators but the mechanism/apparatus for live beam rotation. Heavy-ion gantries are expensive but powerful scientific instruments, and their clinical accessibility is relatively scarce world-wide. Most facilities operate with fixed-beam treatment rooms and only 2 heavy-ion gantry systems are in clinical operation—in addition to our institution, the light compact gantry with superconducting technology at NIRS in Chiba, Japan^57^ began treating patients in 2017. Because most centers equipped with heavy ions are limited to a fixed-beam delivery approach, table rotation around isocenter may also be of interest for arc. For instance, work is underway at the Shanghai Proton and Heavy Ion Center to develop an isocentric rotating chair positioner (patient upright), which could also be used for testing a “pseudoarc” delivery for patients with H&N cancers.^58^ Gantry system requirements for SHArc-C are indeed more costly due to immense size (~700t) and technical challenges. On the other hand, SHArc-He may be more practical for immediate widespread application since arc delivery would be functional for a smaller gantry system nearly equivalent to common proton therapy systems.

**Figure 7:**
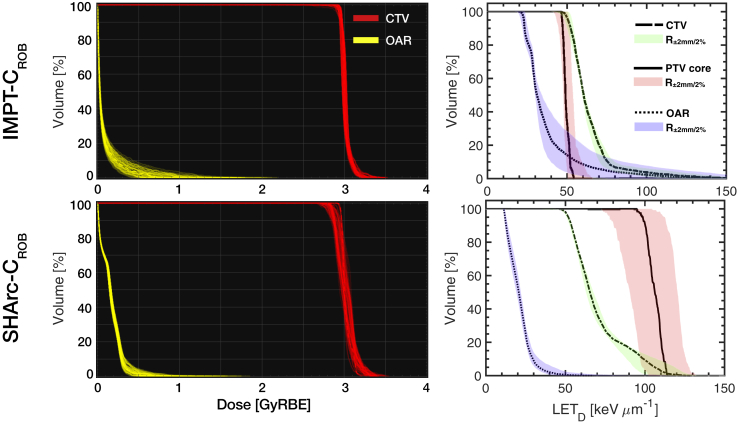
Effect of 3-dimensional (3D) patient positioning ($\vec{r}$) and range (R) uncertainty on relative biological effectiveness (RBE)-weighted dose and dose-averaged linear energy transfer (LET_D_) distribution for ^12^C using intensity modulated particle therapy (IMPT)-2F (conventional) and spot-scanning hadron arc (SHArc) delivery. Dose-averaged linear energy transfer volume histogram (LET_D_VH) profiles for the planning target volume (PTV) core and organ at risk (OAR) are provided considering $\vec{r},$R ± 2%. LET_D_ robustness window is represented by shaded regions (green = clinical target volume [CTV], red = PTV core, blue = OAR). (A color version of this figure is available at https://doi.org/10.1016/j.adro.2021.100661.)

Previous works suggest proton arc could potentially reduce effect of range uncertainty and improve target conformality compared with photon beams for thoracic treatments^59^; however, variable RBE and its effect end-of-range were not considered, which may hinder overall treatment robustness. Investigations with helium and carbon ion arc delivery are expected to produce similar results based on observations in this study, but due to great RBE variations, investigation of plan robustness is critical to determine reliability of the delivery technique. To address these concerns, additional SHArc treatment optimizations using robust planning (SHArc_ROB_) were performed on case B. Angular-dependent beam energy modulation was conducted to displace ranges by ±6 mm from the nominal energy (BP at target center) in steps of 2 mm (1 nominal and 6 modulated). The 7 energies generated subarcs with 14° intervals. Here, the nominal energy was maintained from case B (218.52 MeV/u for ^12^C ions), but in theory, each subarc can have a different nominal energy depending on patient/phantom geometry. Optimization protocols otherwise followed the same procedure as outlined in the Methods and Materials section but applied robust planning objectives accounting for 3-dimensional patient positioning ($\vec{r}$) and range (R) uncertainty effects, using ±3%/3 mm $\vec{r}$/R criteria (21 scenarios).^60^ Resultant robust optimizations for IMPT-C and SHArc-C plans are presented in Figure 7, yielding clinically acceptable uncertainty in delivered biological dose to the clinical target volume (CTV) for SHArc (±6%) compared with IMPT-2F (±3%), with 74% and 99% of voxels passing within ≥95% of prescription dose (2.85 GyRBE) for D_95%,CTV_ and D_90%,CTV_, respectively. SHArc_ROB_ produced alterations in LET focusing compared with SHArc using a monoenergetic beam energy (Figs 3 and 4), with a decrease in LET_max_ in the PTV core from ~150 keV·μm^-1^ to ~120 keV·μm^-1^. Despite the LET_max_ reduction, ~60% volume increase in high-LET components >100 keV·μm^-1^ was observed for SHArc_ROB_. Considering combative LET levels for hypoxia begin around ~100 keV·μm^-1^ (Fig 2), these changes in LET_max_ would have minor effects on HRF mitigation, and the increased volume of LET > 100 keV·μm^-1^ would play a greater role in increasing TCP than LET_max_.

With regard to OAR sparing, SHArc_ROB_ exhibited a 74% passing rate for voxels ≤1 GyRBE in 0.03 cm^3^ from the nominal plan compared with robust IMPT-C’s 36% passing rate (among 70 scenarios for robustness evaluation applying conditions within ±2%/2 mm $\vec{r}$/R). Moreover, SHArc_ROB_ planning substantially reduced LET levels in the OAR (LET_2%_ of 43 keV·μm^-1^ vs 136 keV·μm^-1^ for SHArc and IMPT, respectively) and uncertainty (shaded area in Fig 7). Preliminary results indicate robust SHArc plans may be feasible, deliverable, and of clinical value.

As demonstrated in the published SPArc method,^5^ robustness is made possible for proton beams with dedicated arc optimization protocols via multienergy layer switching. In this work, we demonstrated that robust plans can be effectively optimized with high LET centralized in the target core using both light and heavy ions accounting for variable RBE. In general, a greater range of energies used during arc optimization will lead to a more robust plan but will simultaneously diminish the centralized high-LET components. Therefore, the balance between robustness and enhanced treatment features is crucial for SHArc, and investigations continue to determine optimal optimization protocols for heavier ions. Here, the initial focus was on carbon ions due to potential challenges in robust optimization with the greater RBE/LET gradients and uncertainties compared with lighter ions. Nonetheless, monoenergetic proton arc techniques have yet to address robustness of the LET distribution and effect on biophysical distributions.^13^

In summary, one must note that for arc delivery with heavy ion beams, small variations in range and positioning can affect the centralized high LET region if not optimized robustly. Clinically relevant uncertainties may otherwise induce LET variations, and thus, changes in biological dose, particularly an issue for RBE-weighted dose optimization planning as performed with heavy ions. Moreover, selected tissue type has been shown to greatly affect LET/dose-dependent trends,^31^ and other tissue models may exhibit lessened bio-dose sensitivity to transposed high-LET regions (ie, higher α/β and smaller charged particles). In sum, compared with the nominal plans, SHArc may deliver slightly less homogeneous target doses (elevated core dose), a prospective tradeoff with greater certainty in OAR dose and reduced high-LET components in the OAR. Biological dose robustness does not account for hypoxia factors (Fig 7) and therefore, a reduced target dose homogeneity may be desirable considering LET boosts in the target core.

Recent works in the literature on radiobiological effects of proton monoenergetic arc therapy (PMAT) versus conventional IMPT do not report robustness; however, considering the results of this work, it is likely that single energy arc delivery without explicit robust optimization may be inherently susceptible to range and set-up uncertainties, with a resultant increase in biological uncertainty. To mitigate these effects, stereotactic delivery with robust optimization should be considered in future developments.

New-age CT systems can significantly diminish these systematic uncertainties in range (±1%) during Hounsfield Unit (HU)-to-stopping power conversion and could prove useful in complex scenarios.^61^^,^^62^ Nonetheless, the degree of the intrinsic range/bio-effect uncertainty in patients and suitable prescription doses for SHArc treatments must be considered in future works.

Proton therapy is becoming a widespread radiation therapy technology with several well-established vendors, and accordingly, SPArc/PAT techniques are a principle clinical interest with plans for large-scale commercialization underway in the form of academic/industry collaborations with IBA (Ion Beam Applications, Louvain-la-Neuve, Belgium) and RaySearch (Stockholm, Sweden). Proton distributions theoretically provide ideal characteristics for arc delivery, particularly in the vicinity of critical structure, which requires strict dose avoidance. As a result, dosimetric improvements of proton arc compared with conventional IMPT are well documented in the literature for H&N, prostate, and thoracic cancers. Nonetheless, our results demonstrate that for the investigated conditions with our facility settings, the enhancement in bio-effect, tumor targeting (ie, reduced multiple coulomb scattering), and distal fall-off with heavier ions outperformed proton beams in monoenergetic arc delivery, despite spallation processes and subsequent fragmentation tail extending beyond the BP (increasing with particle M and Z) into surrounding normal tissues. Our facility has unique proton beam characteristics and beam application and monitoring systems composed of high Z material (eg, tungsten) compared to modern systems with short nozzle to isocenter distances. Accordingly, these conditions adversely affect the pristineness of lower Z particle beams. Therefore, succeeding works to study SHArc-p with more common beamlines, as well as in the context of performance against its predecessors SPArc and PAT, are warranted. With nearly 13 centers in operation with carbon ions (and potentially ^4^He), heavy-ion therapy is on the rise and the findings in this work plainlydemonstrate key dosimetric/biophysical advantages of cutting-edge delivery techniques like SHArc. Investigations are ongoing into the benefits of higher-LET particles with Z > 6 (ie, ^16^O/^20^Ne) using multi-ion and/or SHArc delivery techniques.

For the tested conditions in this study, optimization with a discrete energy arc was sufficient for proper target coverage; however, in practice, several partial arcs of differing beam energies, as shown with SHArc_ROB_, would be required.^10^ More clinical-like planning will involve advanced optimization of various tumor arrangements/shapes and patient geometry. SHArc is by no means an all-in-one treatment solution. For instance, shallow tumors benefit highly from particle therapy by taking advantage of beam angle selection with no or minimal exiting dose (fragmentation tail). If the tumor location is asymmetrical (closer to skin surface on one side), conventional IMPT or partial arc may provide similar or more desirable distributions than full arc delivery, and in such cases, complexity in delivery should be minimized when feasible. Nonetheless, the symmetrical design of the investigated cases has provided ideal “base-line” conditions for SHArc optimization for centrally located tumors.

The main aims of this study were to identify physical, biological and clinical benefits of SHArc to further justify development of hadron arc techniques. Machine limitations and delivery specifications for arc techniques with the heavy ion gantry are currently unresolved, which calls for more technical exploratory investigations and dosimetric comparisons. Additionally, dedicated optimization algorithms for SHArc patient treatments must be finalized for proper clinical assessment. In turn, the next steps involve SHArc optimization within a patient cohort study, essential to systematically identify and evaluate site-specific cases where SHArc treatment techniques are clinically beneficial. For each particle beam, investigation of both tumor control enhancement and effect on secondary cancer induction probability in normal tissues from the low-dose bath should be examined. Development and clinical evaluation of robust treatment planning and delivery techniques is progressing for SHArc at our facility to improve treatment efficacy in particle therapy.

## Conclusion

We propose the first arc treatment technique using helium and carbon ion beams and provide evidence in silico that SHArc therapy may offer uniquely valuable clinical advantages both dosimetrical and biological. Through arc delivery of high-LET particle beams, enhanced bio-effect is delievered, increasing toward the tumor core and a low-dose bath is delivered to surrounding healthy tissues. In turn, robust SHArc treatments could potentially improve tumor control by overcoming tumor microenvironment resistance factors such as hypoxia-induced radio-resistance and reduce toxicity in critical structures by minimizing high-LET components.
