# Supplementary material for: Spot-Scanning Hadron Arc (SHArc) Therapy: A Study With Light and Heavy Ions
Source: Adv Radiat Oncol. 2021 Feb 4;6(3):100661. doi: 10.1016/j.adro.2021.100661 (PMC8010580; doi:10.1016/j.adro.2021.100661)
Supplement: Supplementary material [file mmc1.pdf]

Supplementary Information to "Spot-scanning Hadron Arc (SHArc) Therapy: a study with light and heavy ions"

Summary

In this work, we have developed a phenomenological approach to predict the oxygen enhancement ratio (OER) for protons, helium and carbon ion beams based on the experimental data available in the literature. The model explicitly accounts for the mixed radiation field spectra (energy and particle species). The OER calculation approach has been implemented in the GPU-accelerated effective dose engine FRoG (Fast Robust dose* engine on GPU) and subsequently the research treatment planning system PRECISE (PaRticle thErapy using single and Combined Ion optimization StratEgies). In this document, we outline the development and mechanics of the OER model in the context of dose- or LET-painting for spot-scanning hadron arc (SHArc) therapy.

Modeling $HRF_{ion}^{O_2}$

In summary, the generalized approach to modeling i.e. $(Z_{eff}/\beta)^2$ using proton, helium and carbon ion data, was applied throughout the study (Fig.1). Table 1 lists extracted data used for model development.

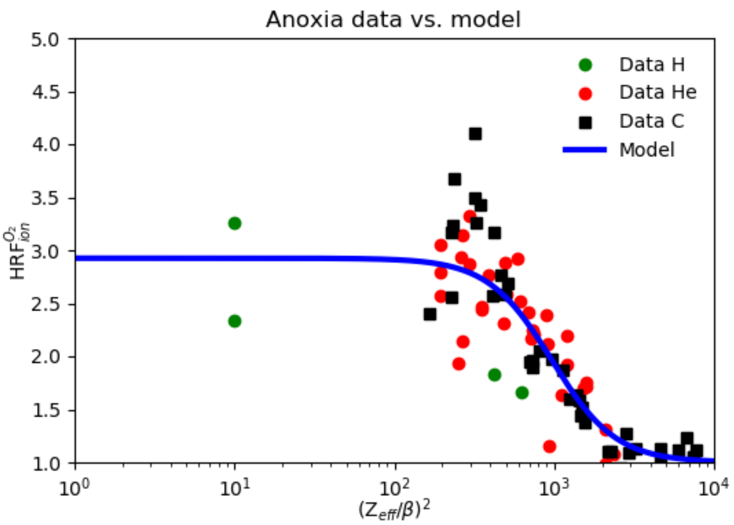

Figure 1. Collected experimental $HRF_{ion}^{O_2}$ data for carbon ions (black square), helium ions (red points) and protons (green points and stars) as function of $(Z_{eff}/\beta)^2$ as reported in the legend. The model has been fitted to the set of data at pO_2 = 0.001% (solid line). The basis of the model presented here was selected for further study due to its applicability to the spectra of ions observed in the mixed-radiation field for carbon ion beams (i.e. proton, helium, lithium, beryllium, boron and carbon ions) and subsequently incorporated into the PRECISE TPS. Dependence on pO_2 is described in following sections.

Cell Line	Particle type	LET [keV/μm]	αh (Gy-1)	βh (Gy-2)	αN (Gy-1)	βN (Gy-2)	O2%	Reference
V79	He	110	1.32	0	1.31	0	<0.0006	Prise *et al* (1990)
human kidney T1	He	110	1.393	0	1.797	0.013	<0.005	Barendsen *et al* (1966)
human kidney T1	He	88	0.73	0.041	1.203	0.136		
human kidney T1	He	61	0.639	0	1.523	0		
human kidney T1	He	26	0.239	0.008	0.972	0		
V79.4	He	120	1.4318	0	1.5435	0	<0.0003	Jenner *et al* (1993)
V79	He	18.6	0.099	0.0042	0.225	0.0426	0.001	Furusawa *et al* (2000)
V79	He	18.6	0.087	0.0051	0.297	0.0317		
V79	He	23	0.165	0.0022	0.255	0.0401		
V79	He	23.8	0.079	0.0064	0.261	0.0569		
V79	He	24	0.108	0.0059	0.168	0.0591		
V79	He	29.9	0.062	0.0118	0.162	0.0676		
V79	He	29.9	0.157	0.0067	0.376	0.044		
V79	He	38.1	0.197	0.0175	0.534	0.0537		
V79	He	39.2	0.132	0.0145	0.529	0.0465		
V79	He	39.4	0.134	0.0175	0.486	0.0471		
V79	He	50	0.153	0.0348	0.561	0.1081		
V79	He	51.9	0.177	0.0305	0.405	0.1327		
V79	He	52.3	0.245	0.0194	0.496	0.1257		
V79	He	61.9	0.329	0.0558	1.04	0.0793		
V79	He	74.6	0.271	0.0819	1.105	0.1413		
V79	He	74.6	0.303	0.0823	0.822	0.184		
V79	He	90.8	0.541	0.0612	0.956	0.1846		
V79	He	90.8	0.565	0.0613	1.117	0.1039		
HSG	He	18.5	0.213	0.0048	0.543	0.0625		
HSG	He	23.6	0.234	0.0066	0.662	0.0692		
HSG	He	32.4	0.442	0	1.221	0		
HSG	He	46	0.557	0.0102	1.328	0.1016		
HSG	He	53.8	0.818	0.0005	1.705	0.0509		
HSG	He	70.3	1.248	0.0433	2.283	0		
P-388	He	26	0.014	0.002	0.063	0.005	<0.002	Berry (1970)
P-388	He	45	0.013	0.001	0.045	0.005		
P-388	He	62	0.066	0.002	0.056	0.013		
P-388	He	86	0.068	0.017	0.022	0.035		
V79	p	17	0.795	0	1.73	0	<0.0006	Prise *et al* (1990)
V79	p	24	0.222	0.007	0.335	0.059		
V79	p	32	0.32	0.041	0.538	0.111		
H4	p	1.21	0.071	0.004	0.273	0.022	0.001	Williams *et al* (1978)
HD1	p	1.21	0.041	0.002	0.076	0.021		
HSG	p	1.2	0.09	0.0059	0.19	0.059	<0.026	Kanemoto *et al* (2014)
HSG	p	2.18	0.11	0.0063	0.17	0.066		
HSG	p	2.64	0.08	0.0076	0.15	0.067		
HSG	p	5.75	0.1	0.0076	0.24	0.064		

Table 1. List of experimental data used for developing the HRF approaches for proton and ^{4}He ion beams. The cell line, the particle type (p or He), the LET and the LQ terms in normoxic and hypoxic conditions are reported together with the experimental/assumed O_2 level and related references. The data refer typically to extreme hypoxic conditions (except the Kanemoto et al. data) with O_2 levels lower than ~ 0.002%. For cases where LQ parameters in normoxia and hypoxia were reported, data were extracted directly as opposed to sampling for plots/figures or calculated from other related published quantities. More specifically regarding the former, when only survival curves were available, experimental points and corresponding error bars were extracted digitally

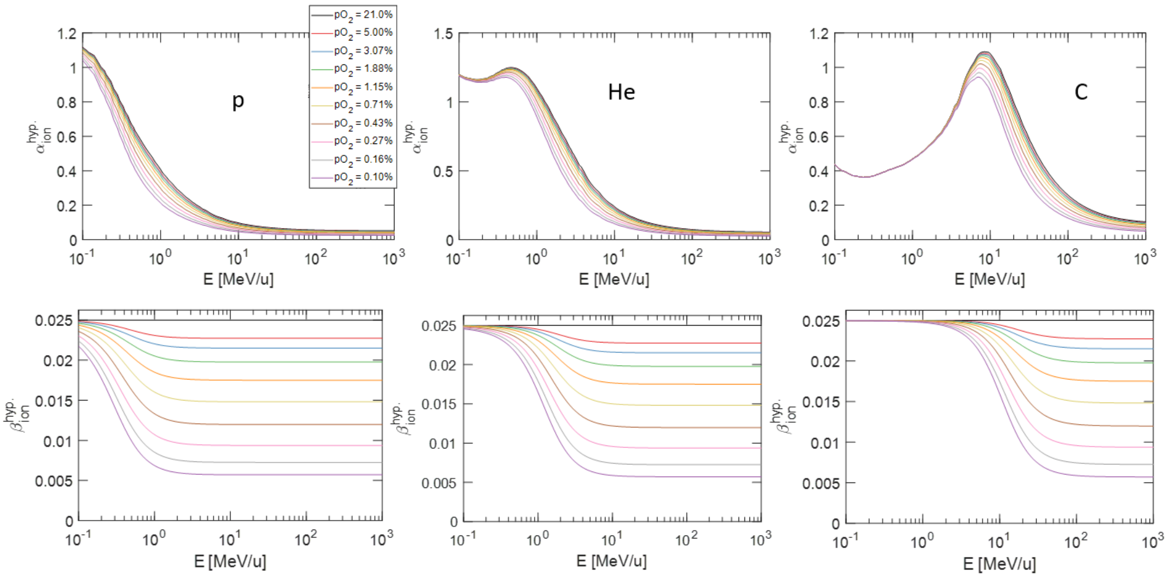

Figure 2. For the three investigated ions (p, ^{4}He and ^{12}C), α_{ion} and β_{ion} as a function of beam energy (MeV/u) for a clinically relevant levels of [O_2] for $(\alpha/\beta)_x$ = 2 Gy using the modified dosimetric kinetic model (mMKM)

RBE Modeling

Cell survival and relative biological effectiveness (RBE) modeling

Biological dose prediction is outlined here based on prior works with carbon ions (Mein *et al.*, 2020). Conventionally, modeling is based on modeling survival (S) of an irradiated cell population within the linear-quadratic (LQ) framework:

$$S = e^{-(\alpha D + \beta D^2)} \quad (5)$$

where α and β represent the linear and quadratic coefficients, respectively, as a function of physical dose (D). The ratio of the linear (α) and quadratic (β) coefficients, $(\alpha/\beta)_x$, the tissue fractionation (Fx) parameter, is often referred to as a description for the sensitivity of the cell line when exposed to a reference photon radiation (x).

To relate biological damage efficacy for particle beams to reference radiation, relative biological effectiveness (RBE), a multi-functional parameter, is defined:

$$\mathrm{RBE}\left[\left(\frac{\alpha}{\beta}\right)_x, \mathrm{LET}, \mathrm{D}\right] = \frac{D_x}{D} \quad (6)$$

RBE represents the iso-effective ratio between a particle radiation (D) and a reference radiation (D_x) and is conventionally modeled with three main dependencies: $(\alpha/\beta)_x$, linear energy transfer (LET) and D. From here, the effective (biological) dose can be computed as the product of RBE and:

$$D_{RBE} = \mathrm{RBE} \cdot D \quad (7)$$

Modeling biological effectiveness of ion beams

Determination of RBE dependencies on photon and particle ion LQ-parameters is performed via consideration an absorbed dose D for an ion beam (with charge Z) and photon dose D_x as iso-effective:

$$\alpha D + \beta D^2 = \alpha_x D_x + \beta_x D_x^2 \quad (8)$$

Solving for RBE:

$$\mathrm{RBE}(\alpha, \beta, \alpha_x, \beta_x) = \frac{\alpha + \sqrt{\alpha^2 + 4\,\beta D_x(\alpha_x + \beta_x D_x)}}{2(\alpha_x + \beta_x D_x)} \quad (9)$$

Two ratios are defined as in (Mairani, Dokic, *et al.*, 2016; Mairani, Magro, *et al.*, 2016):

$$\mathrm{RBE}_\alpha \equiv \frac{\alpha}{\alpha_x} \quad (10)$$

$$R_\beta \equiv \frac{\beta}{\beta_x} \quad (11)$$

Equation 9 can be written with equations 10 and 11 as:

$$\mathrm{RBE}\left(\left(\frac{\alpha}{\beta}\right)_x, D_x, \mathrm{RBE}_\alpha, R_\beta\right) = \frac{\left(\frac{\alpha}{\beta}\right)_x \mathrm{RBE}_\alpha + \sqrt{\left(\frac{\alpha}{\beta}\right)_x^2 \mathrm{RBE}_\alpha^2 + 4D_x\left[\left(\frac{\alpha}{\beta}\right)_x + D_x\right] R_\beta}}{2\left[\left(\frac{\alpha}{\beta}\right)_x + D_x\right]} \quad (12)$$

Here, RBE dependencies on photon (i.e. $(\alpha/\beta)_x$ and D_x) and ion parameters (i.e. RBE_α and R_β) are made evident. Derivation as function of ion dose D yields:

$$\mathrm{RBE}\left(\left(\frac{\alpha}{\beta}\right)_x, D, \mathrm{RBE}_\alpha, R_\beta\right) = -\frac{1}{2D}\left(\frac{\alpha}{\beta}\right)_x + \frac{1}{D}\sqrt{\frac{1}{4}\left(\frac{\alpha}{\beta}\right)_x^2 + \mathrm{RBE}_\alpha\left(\frac{\alpha}{\beta}\right)_x D + R_\beta D^2} \quad (13)$$

In following sections, RBE_α and R_β within the mMKM formalism are presented. One must note that a mixed-field radiation spectra must be considered (e.g. via MC simulation) to comprehensively define bio-effect considering all dependencies of interest: $(\alpha/\beta)_x$, LET/particle energy, particle species and dose.

The modified Microdosimetric Kinetic Model (mMKM)

In the modified MKM (Inaniwa *et al.*, 2010; Mairani *et al.*, 2017), RBE_α is expressed in terms of the saturation-corrected dose-mean specific energy of the domain delivered in a single event z_{1D}^* as follows:

$$\mathrm{RBE}_\alpha = 1 + \left(\frac{\alpha}{\beta}\right)_x^{-1} \cdot z_{1D}^* \quad (14)$$

z_{1D}^* depends on z, the specific energy, and z_{sat}, the saturation-corrected specific energy (accounting for decrease of RBE due to the over-kill, i.e. saturation effects, for high specific energy values (Kase *et al.*, 2006). z and z_{sat} depend on the radius of the domain and cell nuclear, R_d and R_d, respectively (Inaniwa *et al.*, 2010). Best fit values for $R_d = 0.3\ \mu m$ and $R_n = 3.6\ \mu m$ were applied as outlined in the main text, previously obtained by fitting *in vitro* data with ^{1}H and ^{4}He ion beams (Mairani *et al.*, 2017). R_β is expressed as (Kase *et al.*, 2008):

$$R_\beta = 1. \quad (15)$$

Effective dose inputs involve reference photon tissue fractionation parameter $(\alpha/\beta)_x = 2$ Gy with $\alpha_x = 0.05$ Gy^{-1} and $\beta_x = 0.025$ Gy^{-2}. After generation of z_{1D}^* tables as function of beam energy (MeV/u), α_{ion}^{mkm} and β_{ion}^{mkm} values were determined from equations (14) and (15), and subsequently normalized by determined $HRF_{ion}^{O_2}$ values from equation (4). Input tables are plotted in Figure 4 for visualization of trends as a function of beam energy.

Biophysical databases for mMKM-based D_{RBE} calculations (α_{ion}^{mkm} and β_{ion}^{mkm} as a function of depth) in normoxic and hypoxic conditions were generated for all 255 energies available at †INSTITUION-XXX for the three investigated particle species. Figure 2 displays α_{ion}^{mkm} and β_{ion}^{mkm} trends for various pO_2 levels. The effective dose prediction can then be computed accounting for impact of oxygenation level as follows:

$$D_{OER.RBE} = \sqrt{\frac{ln(S_{OER})}{\beta_x} + \left(\frac{\alpha_x}{2\beta_x}\right)^2} - \left(\frac{\alpha_x}{2\beta_x}\right) \tag{16}$$

where S_{OER} = $exp\,[-1 * (\alpha_{ion}^{mkm}([O_2])D^2 + \beta_{ion}^{mkm}([O_2])D^2)]$, the survival of the specified cell line under hypoxic conditions with a given $[O_2]$. $D_{OER,RBE}$ underlines that calculations do not directly involve RBE-weighted dose, as only the particle beam parameters account for hypoxia (that is, photon parameters α_x and β_x corresponding normoxic photon radiosensitivity parameters).

References

Inaniwa, T. *et al.* (2010) 'Treatment planning for a scanned carbon beam with a modified microdosimetric kinetic model', *Physics in Medicine and Biology*, 55(22), pp. 6721–6737. doi: 10.1088/0031-9155/55/22/008.

Kase, Y. *et al.* (2006) 'Microdosimetric measurements and estimation of human cell survival for heavy-ion beams', *Radiat. Res.*, 166(4), pp. 629–638. doi: 10.1667/RR0536.1.

Kase, Y. *et al.* (2008) 'Biophysical calculation of cell survival probabilities using amorphous track structure models for heavy-ion irradiation', *Physics in Medicine and Biology*, 53(1), pp. 37–59. doi: 10.1088/0031-9155/53/1/003.

Mairani, A., Dokic, I., *et al.* (2016) 'Biologically optimized helium ion plans: calculation approach and its in vitro validation', *Physics in Medicine and Biology*, 61(11), pp. 4283–4299. doi: 10.1088/0031-9155/61/11/4283.

Mairani, A., Magro, G., *et al.* (2016) 'Data-driven RBE parameterization for helium ion beams', *Physics in Medicine and Biology*, 61(2), pp. 888–905. doi: 10.1088/0031-9155/61/2/888.

Mairani, A. *et al.* (2017) 'Optimizing the modified microdosimetric kinetic model input parameters for proton and 4 He ion beam therapy application', *Physics in Medicine and Biology*, 62(11), pp. N244–N256. doi: 10.1088/1361-6560/aa6be9.

Mein, S. *et al.* (2020) 'Assessment of RBE-weighted dose models for carbon ion therapy towards modernization of clinical practice at HIT: in vitro, in vivo and in patients', *International Journal of Radiation Oncology*Biology*Physics*. doi: 10.1016/j.ijrobp.2020.05.041.
